# Understanding the Behavior of Sodium Polyacrylate in Suspensions of Silica and Monovalent Salts

**DOI:** 10.3390/polym15193861

**Published:** 2023-09-22

**Authors:** Gonzalo R. Quezada, Francisco Retamal, Matías Jeldres, Ricardo I. Jeldres

**Affiliations:** 1 Escuela de Ingeniería Civil Química, Facultad de Ingeniería, Universidad del Bío-Bío, Concepción 4030000, Chile; francisco.retamal2002@alumnos.ubiobio.cl; 2 Departamento de Ingeniería Química y Procesos de Minerales, Facultad de Ingeniería, Universidad de Antofagasta, Antofagasta 1240000, Chile; hugo.jeldres.valenzuela@ua.cl (M.J.); ricardo.jeldres@uantof.cl (R.I.J.)

**Keywords:** sodium polyacrylate, quartz, rheology, molecular dynamics, monovalent salt

## Abstract

This study investigated the interaction of monovalent cations with different sizes on quartz surfaces and the rheological impact that this causes in concentrated suspensions when subjected to the action of a rheological modifier, in this case, sodium polyacrylate (NaPA). Yield stress was determined using a rheometer with a vane-in-cup configuration to establish the relationship between shear stress and strain. Experiments were carried out in LiCl, NaCl, KCl, and CsCl solutions. The results show that the yield stress increases following the order Li < Na < K < Cs in the absence of PAA. However, the addition of NaPA significantly reduced the yield stress in all cases. This reduction was more noticeable in the LiCl and NaCl solutions than in the KCl and CsCl solutions, suggesting a more pronounced effect of PA in maker salts. We conducted molecular dynamics simulations to understand how PA interacts with dissolved salts on the quartz surface. Our results showed that Li had the highest adsorption, followed by Na, K, and Cs. As the salt concentration increased, so did the adsorption. We validated these simulation results with rheological experiments, which helped us understand the observed differences. The molecular interactions indicate that, in the lithium system, cationic bridges and the synergy between hydrogen bridges and hydrophobic bridges predominate mainly. This tendency decreases as the type of cation is changed due to the decrease in the electrical density of the cation in the following order: Li < Na < K < Cs. This reduces bridging with the quartz surface and, therefore, directly impacts the system’s rheological properties.

## 1. Introduction

Mine tailings have become a concern within the mining industry due to the complex management requirements and the vast array of issues they encompass, including the characterization of essential water resources for mining activities, the intricate storage challenges posed by reservoirs, and, notably, the substantial environmental challenges entailed in their oversight. Some tailings are disposed of as pastes, a highly efficient alternative for recovering water in the tailings and for environmental preservation. The tailings undergo a thickening process, increasing the solid concentration to values near 55–70 wt% to generate the thickened slurry. The resulting suspension must then be transported by pumping and, on certain occasions, aided by gravity and shear-thinning strategies that reduce the rheological parameters of the suspension. The rheological behavior is intrinsically linked to its composition, which influences the critical consistency threshold of the tailings. Beyond this threshold, a notable increase in viscosity and yield stress is observed, a determinant aspect of the material’s flow behavior.

To enhance the flowability of mining tailings, dispersant additives may be employed. These chemical substances play a key role in greatly facilitating the handling and transportation of tailings. Their function revolves around diminishing the attractive forces operating among the solid particles in the tailings, achieved through applying mechanisms such as electrostatic repulsion and/or steric hindrance [1]. This effect enables the uniform dispersion of the particles in suspension, contributing to the sustained stability of the said suspension. The selection of the most appropriate dispersant additive becomes crucial and is intricately tied to the specific characteristics of the given tailings. Aspects such as mineralogical composition, particle size, and pH level directly influence this choice. From this standpoint, it is possible to reduce the viscosity of the suspension through the precise inclusion of a dispersant additive, taking advantage of specific molecular interactions that induce effective particle dispersion.

An alternative involves the application of a polyacrylic acid such as sodium polyacrylate (NaPA), which is a low molecular weight polymer with properties and a chain length that can be modified through varying reaction conditions [2]. This polymer has an interesting behavior that can be used in many applications [3,4,5,6,7], including in the lithium industry [8] and tailing treatment in mining operations [9,10]. In the context of mineral processing, it finds common usage in the mining industry as a dispersant additive for enhancing the flotation stage’s efficiency and improving tailings fluidity. NaPA is water-soluble; its salts are neutralized at molecular masses up to 100,000 g/mol [11,12,13]. This constitutes a fundamental consideration, given its significant impact on rheological properties and dispersion behavior. Consequently, exerting control over molecular weight distribution is dominant. It is imperative to acknowledge that contemporary mining challenges encompass seawater utilization [14,15,16]. Consequently, when examining dispersant applications, behavior in the presence of salts becomes a requisite study, given the diverse range of ions present in low-quality waters. Under such conditions, system complexity escalates, making it imperative to investigate this matter using methodologies capable of elucidating molecular-scale phenomena.

Molecular dynamics emerges as a fundamental tool for comprehending and elucidating the inherent phenomena associated with mineral processing. Within this context, it is an apt avenue for conducting detailed analyses of polymer adsorption at surface interfaces, affording crucial insights into the polymer conformational structure, adsorption mechanisms, and surface alterations induced by polymer presence. Furthermore, it facilitates in-depth examinations of intermolecular interactions, encompassing Van der Waals forces, hydrogen bonding, electrostatic interactions, and steric repulsions, all of which play a pivotal role in adsorption [17,18,19]. The literature shows notable actions to explore the interfaces between oxides and water. The comprehensive analysis undertaken by Wang et al. [20] combined diverse investigations that have converged in this domain. Concerning quartz, various studies have successfully modeled its surface [21,22,23,24], encompassing scenarios involving the presence of dissolved salts [25,26,27,28,29,30], as well as the incorporation of adjunct molecules [31,32,33,34].

This study focuses on exploring NaPA as a rheological modifier for quartz silica at neutral pH in the presence of various concentrations of salts within an aqueous environment. The investigated salts encompass both structure-making and structure-breaking salts, categorizing ions based on their ability to induce robust reorientation of the surrounding water (“maker”) or to displace water from their immediate environment (“breaker”). The salts include LiCl, NaCl, KCl, and CsCl, with LiCl displaying the highest “maker” character, while CsCl exhibits the most significant “breaker” characteristics. This approach was initially developed through an experimental framework, assessing the tangible impact of NaPA on silica pulps. Subsequently, by employing molecular dynamics, an analysis was undertaken to scrutinize the molecular-level interactions between NaPA and quartz surfaces. This bidirectional approach enabled us to comprehend the effects observed in experimental trials while simultaneously unraveling the underlying mechanisms at the molecular level. The study harmonizes practical observations with an advanced computational approach, thereby enriching our understanding of how NaPA influences quartz silica’s rheological properties and surface interactions within intricate and varied environments.

## 2. Experimental Methodology

### 2.1. Materials

The quartz particles obtained from Donde Capo (Santiago, Chile) were crushed and pulverized until 100% of the sample was obtained under separation with a #370 mesh (prior sieving). The SiO_2_ content was greater than 99% by weight according to X-ray diffraction (XRD) [35]. Figure S1 shows the spectrum of NaPA that highlights the structure of their moieties. NaPA of high purity and a molecular weight of 5000 g/mol was obtained from Sigma-Aldrich (Santiago, Chile). For the infrared analysis of the polymer, a Fourier infrared spectrometer (PerkinElmer, Santiago, Chile) was used in the range of 4000 to 400 cm².

The salts used were analytical chemical grade LiCl, NaCl, KCl, and CsCl alkali metal chlorides provided by Merck, Santiago, Chile.

### 2.2. Rheology

For each test, 100 mL quartz suspensions were prepared with 70% solids *w*/*w* at pH 7 and a chloride salt concentration of 0.1 M (LiCl, NaCl, KCl, or CsCl as appropriate), which were then adequately mixed for 1 h via mechanical agitation at a stirring rate of 400 rpm to ensure particle dispersion. Once the suspension was homogenized, the reagents involved (NaOH and NaPA) were added, and stirring was continued for 5 min. Subsequently, a 45 mL portion was withdrawn and poured into the rheometer sensor (paddle cup). The tests were carried out in an Anton Paar MCR 102 rheometer (ANAMIN Group, Santiago, Chile) using a vane in cup configuration with diameters of 2.2 and 4.2 cm, respectively. The measured data was processed using Rheocompass^TM^ Light software version 1.30 (ANAMIN Group, Santiago, Chile). The sodium ions that form NaOH or NaPA were less than 10^−5^ mol/L and, therefore, were a negligible presence. 

The elastic limit was detected in the logarithmic representation of the strain (γ) over the shear stress (τ). Up to the specific shear stress, the relationship between γ and τ was constant, representing the range of elastic deformation. At the end of this range, irreversible deformation occurred with increasing shear stress, resulting in sample flow and a steeper slope of the curve. To determine the yield strength on the log γ/log τ diagram, the curvature of the measurement curve was analyzed with the help of two tangents applied to the two slopes. The measurement was carried out in a logarithmic time ramp with an initial duration of 60 s and a final duration of 1 s, linearly increasing the value of the shear stress with 1 Pa intervals.

## 3. Computer Simulation

### 3.1. Molecules and Surfaces

The methodology to build molecules follows our previous work [13,36]. To accomplish this, the polymer NaPA was meticulously designed, comprising 48 acrylate monomers (-CH_2_-CHCOO^−^-), which underwent complete dissociation due to the monomers’ pKa of 4.5. The experimental environment maintained a pH of 7. The 48 monomers were given a molecular weight of 3400 g/mol, which is lower than the experimental value of the NaPA used, but the simulation was performed primarily to quantify the adsorption; therefore, it is suitable to use a smaller polymer chain. Concurrently, the selection favored terminal monomers possessing hydrogen terminals, thereby instigating terminal CH_3_ and CH_2_ groups. A syndiotactic configuration was judiciously adopted, ensuring a homogeneous polymer matrix concerning its primary chain. In the case of these simulations, we denominated the NaPA as PA because all the cases were performed with only the studied salt, and as we pointed out in the experiments, the sodium from NaPA and NaOH were negligible.

**Figure 1:**
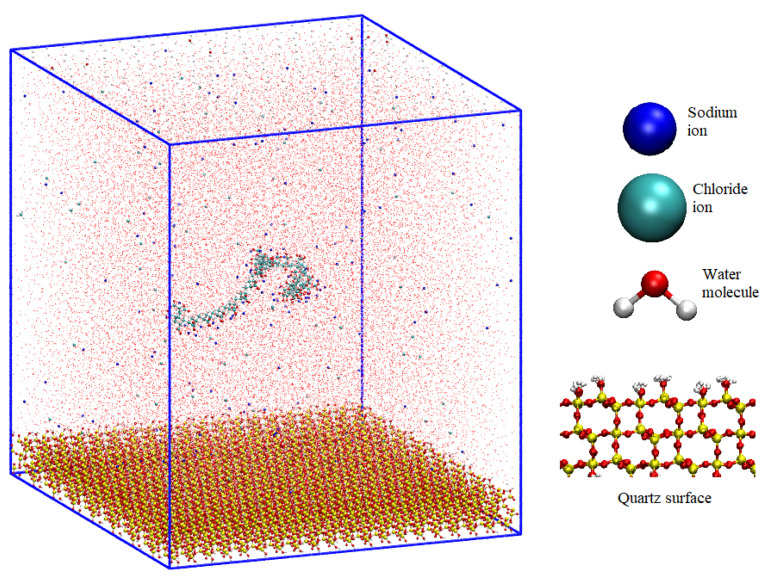
Molecular dynamics configuration box for NaPa with NaCl salt and quartz surface.

The salts under examination, the monovalent LiCl, NaCl, KCl, and CsCl, were delineated via van der Waals spheres, encapsulating both the cations and anions in their essence. The aqueous molecules, embodied by the SPC/E model, defined a triadic depiction, capturing the oxygen atom and its associated hydrogens. For reference of these molecules, see Figure 1.

The substrate of choice for the current analytical work was quartz—a preference that finds roots in our precedent contributions [28,30,33]. The core of the matter resides within the most useful of quartz surfaces, the (101) facet. This, in its intricate interplay, bears emblematic silanol (Si-OH), manifesting in two morphotypes—those prominently juxtaposed with the environment and those transferred to a recessive stance. This dichotomy provides a favorable terrain to elucidate the molecular inclinations against the twin topographies of quartz.

Based on quartz, the structural substrate was made through unit cells with dimensions measuring 0.4916 × 1.3757 × 0.3343 nm^3^. Replication of these units within a 20 × 8 × 3 supercell produced an architectural construct that increased to (Sx × Sy × Sz) = 9.832 × 11.056 × 1.2786 nm^3^. Equilibrium established at pH 7 imparted a negative charge density of −0.03 C/m^2^ on the surface. Furthermore, the more outwardly oriented silanol entities lost a hydrogen moiety in an energetic rearrangement, generating the observed negative charge density. The experimental concentration of the polymer was around 6 × 10^−6^ mol/L, and the simulation was 1 × 10^−3^ mol/L. This might seem like a large difference between the observed and simulation conditions. However, in the simulation, only the solid-liquid interface was considered, so the bulk areas were not studied, and, therefore, the apparent concentration was higher.

### 3.2. Force Field

The potential applied to the PA polymer was the generalized Amber force field (GAFF), which has been previously employed for polymers [37,38]. To acquire this potential, the Antechamber program [39,40] was employed to determine intramolecular potentials, encompassing bonds, angles, dihedrals, and interactions up to 3 atoms apart. The partial charges of each atom were adjusted using the restricted electrostatic potential (RESP) method [41], facilitated by the R.E.D program III.52 [42], integrated with Gaussian 09 [43]. The outcomes of this parametrization for PA were delineated in the work by Quezada et al. [36]. Dissolved ions were modeled according to Li et al. [44], employing the established 12–6 parameters for the oxygen-ion (IOD) distance. The water model adhered to SPC/E ([45]) and was leveraged to ensure geometric constraints for the molecule’s configuration [46]. The quartz surface was derived from CLAYFF [47], supplemented with insights from Kroutil’s work [28] on surface deprotonation. The resultant partial charges have been documented in prior publications [30].

### 3.3. Initial Setup

The initial configuration was generated following the procedures outlined below:

(1) Once the quartz surface with dimensions (Sx × Sy × Sz) was constructed, the dimensions Sx and Sy were employed to define the LX and LY of the simulation box. Subsequently, dimension Lz was established to encompass both the surface and an aqueous solution containing the polymer. Lz was set at 12 nm. Consequently, the simulation box dimensions were (LX, LY, LZ) = 9.832 × 11.056 × 12.000 nm³. The selection of these dimensions is critical to prevent interactions between the polymer and its periodic images.
(2) The polymer was placed in the previously generated space, positioned 5 nm above the surface along the z-direction. This polymer consisted of 48 monomers and had an initial length of 12 nm. Although this length is comparable to the box size, it substantially reduced to less than 5 nm during equilibration stages.
(3) Ions corresponding to the salts investigated in this study were randomly distributed within the space occupied by the flocculant, maintaining a separation of 1 nm between them and the polymer. The concentrations utilized in this investigation were 0.001, 0.01, and 0.1 moles/liter. The total number of cations and anions was determined based on the salt concentration and the counterions necessary to neutralize both the quartz surface and the PA.
(4) Finally, water from an equilibrated configuration at 300 K was introduced, and this configuration was replicated throughout the box until completion. Water molecules that coincided with the preexisting elements in the box were removed. The elimination criterion was based on less than 0.3 nm between the water molecules and other atoms (the initial setup can be seen in Figure 1).

### 3.4. Molecular Simulation

The present study employs classical molecular dynamics (CMD) due to the system’s lack of appreciable reactivity under the investigated conditions [32,48]. Simulations were conducted using the Gromacs 2021.3 simulation software [49]. The ACPYPE program [50] was employed to transfer the force fields obtained in Section 2.2 to the Gromacs format. Execution of the required simulations in this work entails a series of system equilibration steps.

1. Minimization of forces in the initial configuration. This step is essential since the initial configuration stems from stable yet vacant configurations, which necessarily alter upon their assembly. For quartz, solely hydrogen atoms were allowed movement.
2. NVT-equilibration simulation for 0.1 ns. This simulation was conducted at 300 K, with the positions of the polymer and ions held fixed, while only water molecules exhibited motion.
3. NVT-equilibration simulation with annealing for 1 ns. During this simulation, the temperature rapidly increased from 300 to 450 K within 0.0001 ns, followed by a 0.5 ns maintenance at the elevated temperature and then a return to 300 K over 0.5 ns. This annealing process enhanced polymer fluidization in the liquid phase, achieving a stable configuration.
4. NPT-equilibration simulation for 2 ns. Conducted at 300 K and 1 bar pressure, adjustments to the simulation box were confined to the z-direction to relax the pressure to the predefined value.
5. Lastly, an NVT-production simulation spanning 100 ns captured the time evolution of the PA’s interaction with the quartz surface under varying salt concentrations. Six repetitions were performed to mitigate statistical error.

The integration timestep was set at 2 × 10^−6^ ns due to utilizing the LINCS algorithm [51] coupled with hydrogen bond constraints. Temperature control relied on the Nose-Hoover thermostat [52,53] with relaxation times of 0.0025 ns. Pressure control employed the Parrinello-Rahman barostat [54] with relaxation times of 0.001 ns. The cutoff radii for potentials were set at 1.2 nm. The Ewald particle mesh method [55] was utilized for long-range interactions, and Lorentz-Berthelot mixing rules governed cross-interactions.

### 3.5. Data Processing

To ascertain adsorption outcomes, the “gmx mindist” program provided by the Gromacs code was employed. Within this program, a pair-wise atom enumeration was conducted for atoms that exist at minimal distances, in this context, between the polymer and the surface. A minimum interaction distance of 0.5 nm was chosen to account for this interaction between the center of masses.

Our own code was utilized to compute the hydrogen bond (HB) and cationic bridge (CB) interactions [56]. In the case of HB interactions, associations between electronegative groups, mediated by a hydrogen atom bonded to one of the two groups, were quantified within a distance range of 0.24 to 0.3 nm. Concerning CB interactions, the interaction between electronegative groups mediated by an ion dissolved in the system was quantified. In this scenario, interactions involving only oxygen atoms as electronegative groups were pertinent. The bridge calculations were executed at distances of less than 0.3 nm for all utilized salts. 

The calculation of ion adsorption and net charge profiles was carried out using the “gmx density” program from the Gromacs software version 2021.2 suite. The “number” option was selected for ion adsorption to determine atomic density. Conversely, the “charge” option was chosen for charge profiles to quantify the electronic charge distribution across the simulation box.

## 4. Results

### 4.1. Rheological Behavior

The impact of sodium polyacrylate dispersant and salt type on the yield stress of a quartz suspension was examined. To this end, a suspension comprising 68 %tw and a salt concentration of 0.1 M at a pH of 7 was formulated, both in the absence and presence of the dispersant. The controlled dosage of sodium polyacrylate amounted to 200 g/t, ensuring the attainment of the utmost degree of achievable dispersion in each suspension.

**Figure 2:**
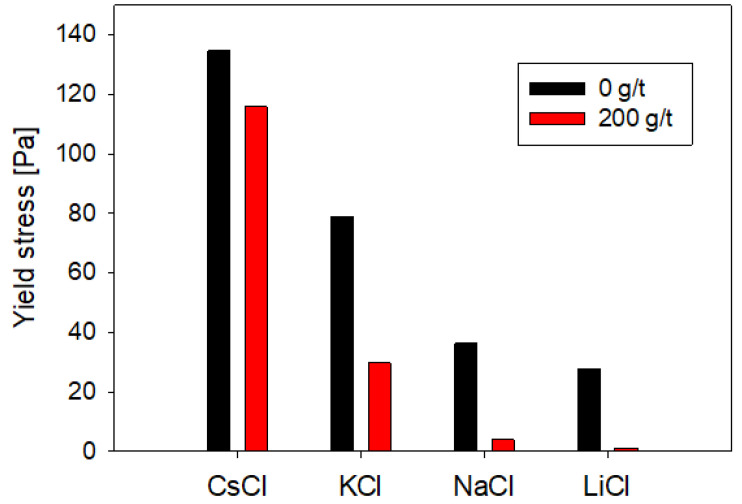
Effect of the type of salt and presence of NaPA on the rheological behavior of quartz suspensions.

As illustrated in Figure 2, the influence of salt type on the pulp’s yield stress manifested as significant. Pulp compositions featuring salts with cations characterized by smaller ionic radii exhibited notably diminished yield stress values compared to those incorporating cations with larger ionic radii. For instance, the suspension in the presence of cesium (ionic radius of 1.69 Å) demonstrated a yield stress of 135 Pa. In contrast, in the presence of lithium (ionic radius of 0.6 Å), the yield stress registered 28 Pa in the absence of the dispersant.

Upon the introduction of sodium polyacrylate, all suspensions recorded a reduction in yield stress attributable to heightened electrostatic and steric repulsion caused by the reagent at the particle surface. However, the extent of dispersion and, consequently, the efficacy of the reagent proved acutely responsive to the specific salt type prevailing within the suspension. The percentage reduction in yield stress was amplified in the presence of cations characterized by diminished ionic radii, culminating in a decrease of 95.8% in the case of lithium, whereas in cesium, the reduction stood at 14.0% (refer to Figure 2).

The outcomes cast light on two intriguing phenomena:

(i) An enlargement in cation size precipitated an elevation in yield stress due to the fortification of interparticle bonding. Considering that quartz lends itself to a conceptualization akin to “breaker” salts in certain aspects, it is foreseeable that pulps experience a more pronounced loss of fluidity in the presence of such salts. This observation aligns with the findings reported by Jeldres et al. [57], who proffered an explanation informed by electrostatic and structural considerations of water behavior to characterize rheological conduct.
(ii) Sodium polyacrylate evinced heightened effectiveness in tandem with smaller salts. The augmented effectiveness can be attributed to its superior adeptness in traversing the hydrated layer enveloping surfaces, particularly in scenarios where the ionic milieu comprises “maker” salts.

### 4.2. Polymer Adsorption

**Figure 3:**
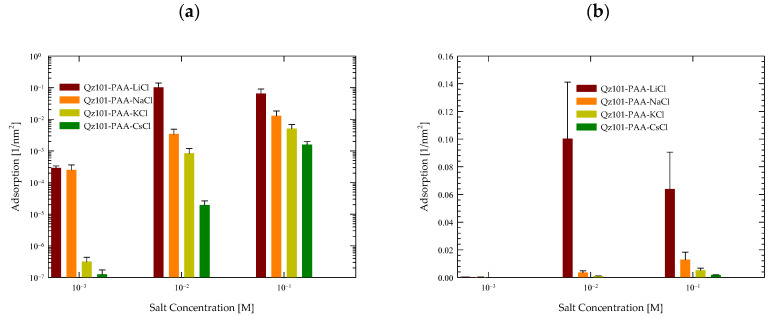
Surface of contacts of PA with quartz (101) surfaces by area at different salt types and with a concentration at pH 7. (**a**) Logarithmic scale. (**b**) Linear scale.

Figure 3 illustrates the quantification of the surface adsorption of quartz minerals across various salts (LiCl, NaCl, KCl, and CsCl) at three distinct concentrations, facilitating an exhaustive comparison across each scenario. The methodology, outlined in Section 3.5, was based on averaging the results from six simulated repetitions.

A systematic augmentation in quartz adsorption emerged as salt concentration increased in all cases. These findings reaffirm the mineral’s surface capacity for adsorption, notwithstanding the limitation imposed by its low charge density, which hinders the effective attraction of ions at low concentrations. However, at higher concentrations, the feasibility of effective quartz adsorption becomes more pronounced.

Analyzing each salt individually in Figure 3a, the prominence of quartz adsorption in the presence of LiCl is notable, both at low and high concentrations. Concerning CsCl, minimal surface adsorption was observed at low concentrations, which intensified significantly with concentration elevation. For KCl, the most pronounced rise in adsorption was observed as concentrations increased. In the case of NaCl, while reasonable adsorption was shown, it was less markedly expanded than in the aforementioned cases.

This behavior pattern is more understandable when data is visualized logarithmically, as previously elucidated. However, to accentuate the genuine contrast of adsorption, Figure 3b is presented. Within it, the conspicuous disparities in adsorption are discerned in the presence of LiCl at concentrations of 0.01 M and 0.1 M, yielding values of 0.1 nm^−2^ and 0.06 nm^−2^, respectively. Conversely, in scenarios featuring NaCl, these values declined more prominently to 0.005 nm^−2^ for 0.01 M and 0.015 nm^−2^ for 0.1 M. For KCl, the values diminish significantly to 10^−3^ nm^−2^ for 0.01 M and 0.01 nm^−2^ for 0.1 M. Ultimately, in the case of CsCl, the values plummeted even further to 10^−5^ nm^−2^ for 0.01 M and 10^−3^ nm^−2^ for 0.1 M. Findings at salt concentrations of 0.001 M yielded negligible values.

Considering our prior investigation (refer to [56]), wherein a comparable analysis was conducted involving PA exclusively with NaCl at different molar concentrations, the current outcomes corroborate prior discoveries and uphold the trend of PA’s response to heightened salt quantities where atom contacts were 2 × 10^−3^ at 0.006 M and 6 × 10^−2^ at 0.6 M of NaCl. This ongoing study evinces that the cationic type exerts discernible effects on outcomes, particularly within LiCl salts. To a lesser extent, a surge in adsorption was noted in the presence of KCl, while adsorption considerably decreased in the presence of CsCl.

These results signify a direct correlation between PA’s affinity with the quartz surface and the size of the accompanying cation. Given PA’s substantial charge and quartz’s weak charge density, the anticipated interaction would inherently be limited. Nonetheless, these results substantiate that the interaction’s nature is significantly influenced by the specific cation present in the system.

**Figure 4:**
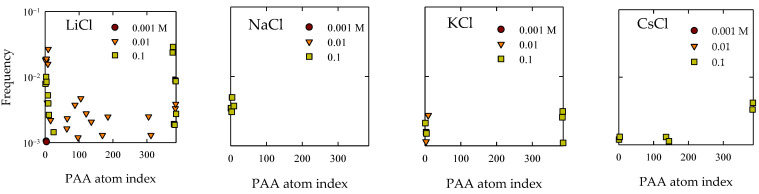
Atomic contact distribution of PA with quartz (101) surfaces at different salt types and with concentrations at pH 7.

An additional approach for studying the adsorption of PA onto quartz surfaces involves a meticulous exploration of contact distribution in relation to the most frequently approaching atoms. This methodology serves as a complement to the comprehensive analysis conducted using Gromacs’ “mindist” program. Figure 4 illustrates the outcomes concerning contact frequency, tethered to the atomic index of the PA polymer. Given its linear nature, this index commences at 1, corresponding to atoms in the first monomer, and extends through the final monomer at index 48.

Significantly, in simulations involving the presence of LiCl salts, it manifested that at a concentration of 0.01 M, adsorptions manifested at sites associated with internal monomers. Upon considering the findings depicted in Figure 4, a discernible prominence is discerned in the number of contacts at 0.01 M compared to 0.1 M, particularly within LiCl. An insightful examination of Figure 4, within the context of this perspective, unveiled that this upsurge aligned primarily with the emergence of these internal sites. Concerning the remaining scenarios, by and large, the presence of adsorptions at terminal groups is discerned. Solely in the case of CsCl at a concentration of 0.1 M, some internal sites affixed to the quartz surface can be identified.

**Figure 5:**
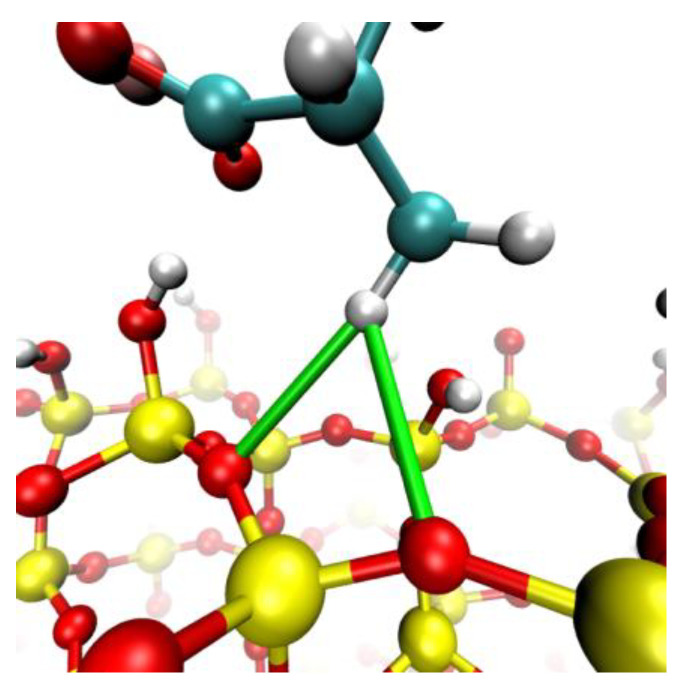
Hydrophobic interaction represented by a green bond obtained in simulation between hydrogen from the CH_3_ moieties and the Si-O-Si moieties.

The culmination of these results solidifies the notion of molecular recognition between the PAA molecular entities and the quartz topography. This interaction materialized as specific adsorption, where a substantive segment of the chain’s exposure to its immediate environment became pronounced with an increase in salt concentration, as can be seen in Figure 4. It is worth accentuating that the prominent atoms approaching the surface are those deprotonated oxygen atoms affiliated with acrylate. Nevertheless, consistent observation across all cases materialized in the propensity for the proximity of the CH_3_ group to the surface (seen in Figure 5). This phenomenon has been documented in antecedent investigatory works [56,58]. This explains a hydrophobic-type interaction, contributing to the adhesive phenomenon between PA and quartz. This interpretation was founded on the consideration that the partial charge borne by the CH_3_ group does not surpass −0.1e, which, in comparison to the charge exhibited by the COO group, quantified around −1e, is significantly diminished. Consequently, it is inferred that this interaction does not prevail over predominant electrostatic forces as concentrations increase. In the case of NaCl, while reasonable adsorption is shown, it is less markedly expanded than in the aforementioned cases.

### 4.3. Polymer Interaction

Hydrogen bonds (HB) and cationic bonds (CB) were scrutinized to probe atom-to-atom interactions. These interactions were tracked over time for each system and categorized based on the atom type on both the surface and polymer. Following the established nomenclature for the quartz surface, we recognize charged or deprotonated silanol oxygens (OM), neutral silanol oxygens (OH), and neutral surface oxygens (OS). For PA, only deprotonated carboxylic oxygens (OP) exist.

**Figure 6:**
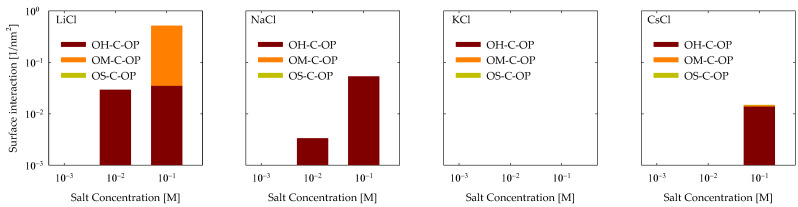
Cation bonds of PA with quartz (101) surface by area at different salt types and with concentrations at pH 7.

The data is presented in Figure 6 for all examined scenarios for cationic bonds. As evident from the graphs, interactions were predominantly observed in the presence of LiCl, with the highest frequency being OM-C-OP interactions at 0.1 M. Also, OH-C-OP interactions were observed to a lesser extent with LiCl, and interactions increased with rising LiCl concentrations. Moving to NaCl, only OH-C-OP interactions were identified, which also displayed an increment with elevated salt concentrations. No interactions were identified for KCl, while CsCl primarily exhibited OH-C-OP interactions at a salt concentration of 0.1 M.

It is imperative to recall that the carboxylic group exhibits significant attraction to cations, thereby instigating the formation of bridges with the surface, predominantly transpiring between a carboxylic group and an adsorbed cation that makes contact with a vacant site on the surface. Consequently, it is reasonable to anticipate that interactions would be more prevalent with vacant sites. In this context, it was observed that this holds for the encountered OH-C-OP interactions. In the case of OM-C-OP interactions, this outcome arose because the charged group on the quartz surface was more exposed to the environment and possessed diminished strength, rendering adsorptions of lithium cations mild. This, in turn, created opportunities for sites to be vacated, facilitating interaction with the PA.

**Figure 7:**
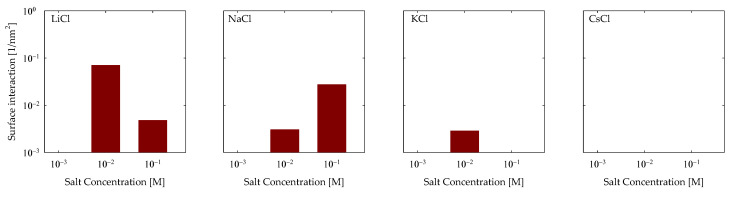
Hydrogen bonds of OH-OP of PA with quartz (101) surface by area at different salt types and with concentrations at pH 7.

Subsequently, an exploration of the hydrogen bonds engendered between the surface and the PA came into focus. The findings from this inquiry are depicted in Figure 7. The outcomes echo analogous trends to those unveiled in the CB analysis, with interactions predominantly manifesting in LiCl and NaCl scenarios. In contrast, a solitary interaction is discerned for KCl and not for CsCl. Aligned with the hydrogen bond taxonomy tailored to PA, only OH-OP type HBs can be delineated due to the polymer’s absence of electronegative hydrogen-bearing groups and the surface’s exclusive endowment of OH groups stemming from silanol functionalities. Notably, within the LiCl context, a heightened quantum of HBs became apparent at 0.01 M, registering at a magnitude of 0.01 nm^−2^, subsequently tapering to 5 × 10^−3^ at 0.1 M. Shifting the focus to sodium, the prevalence of HBs escalated proportionately with NaCl concentration, demonstrating an augmentation from 3 × 10^−3^ to 4 × 10^−2^ nm^−2^. In the realm of KCl, HBs exclusively manifested at 0.01 M, materializing at a magnitude of 3 × 10^−3^ nm^−2^.

**Figure 8:**
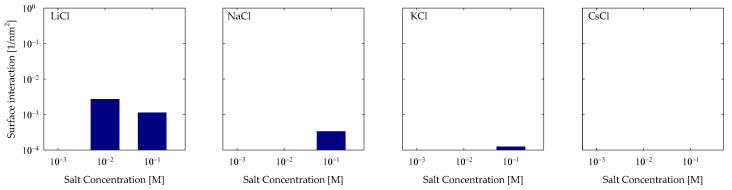
Hydrophobic interactions of OS-C3 of PA with quartz (101) surfaces by area at different salt types and with concentrations at pH 7.

As previously mentioned in Section 4.1 and Figure 5, there is evidence that there are interactions that cannot be classified as cationic or hydrogen bonding. So, we have interactions that fall into the hydrophobic category because the interactions are between groups that have a weak electrical charge; in this case, it was observed that it was between the oxygens of the quartz surface, which are neutralized by their silicon neighbors (OS) and by the hydrogens attached to an aliphatic carbon (C3). To quantify them, a cutoff radius between the OS and C3 groups of 0.5 nm was used. The results can be seen in Figure 8, where, again, a greater trend is seen in maker cations than in breaker cations; this indicates that this interaction is a synergy produced by the HB and CB interactions. Therefore, we see that they keep adding interactions in the case of LiCl and NaCl, while KCl and CsCl have a weak interaction overall.

### 4.4. Ion Adsorption

**Figure 9:**
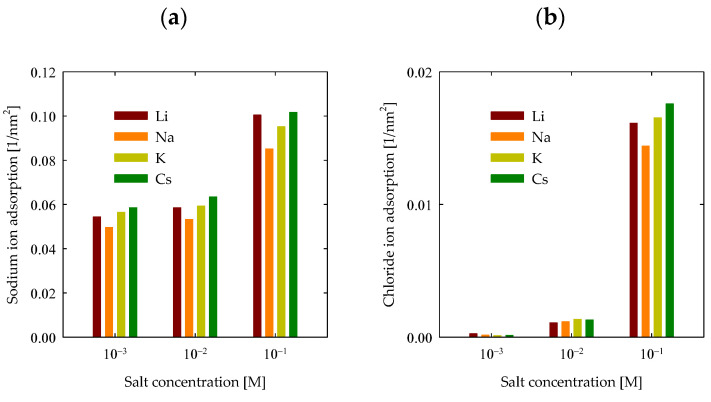
Adsorption of ions with quartz (101) surfaces by area at different salt types and with concentrations at pH 7. (**a**) Na^+^ densities (**b**) Cl^−^ densities.

To proceed with the analysis of PA adsorption on the surface, it is imperative to delve into how ions adhere to the quartz surface. Figure 9a illustrates the adsorption of cations onto the quartz surface. As the salt concentration escalates, a discernible and gradual increase in adsorption becomes evident, transitioning from around 0.05 nm^−2^ at 0.001 M to 0.1 nm^−2^ at 0.1 M. Additionally, it is feasible to discern the impact of the specific cation being adsorbed. It becomes apparent that, except for lithium, adsorption intensifies proportionally with the cation’s size. This behavior implies a partial adherence to the Hofmeister series for these cations. This occurrence primarily stemmed from substantial PA adsorption onto the surface in the presence of LiCl, thereby facilitating the greater adsorption of Li^+^ cations onto the surface. Conversely, the interaction between PA and the surface remained feeble for the remaining cases, henceforth not perturbing the series for Na^+^, K^+^, and Cs^+^ ions. Nonetheless, akin effects were also documented in preceding investigations in the absence of polymers [30], wherein lithium mildly disrupted the series at 0.6 M. Only under alkaline pH conditions did lithium further interrupt the series, as discerned in the mentioned study.

Furthermore, the assessment of chloride adsorption is equally pivotal, with the pertinent results depicted in Figure 9b. At concentrations of 0.001 M and 0.01 M, chlorides on the quartz surface were relatively modest. Quantifying chloride adsorption becomes feasible at 0.1 M, yielding values around 0.02 nm^−2^. The observed trend closely parallels cation adsorption, reinforcing the notion that chloride adsorption is intrinsically tied to cationic presence. This substantiates the proposal that chlorides adhere proximally to cations, manifesting as ion-ion complexes.

### 4.5. Net Charge

**Figure 10:**
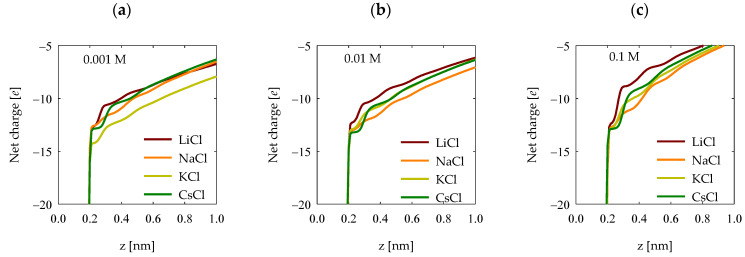
Net charge profiles in the surface of quartz (101) at salt concentrations of (**a**) 0.001 M, (**b**) 0.01 M, and (**c**) 0.1 M.

Quantifying the surface electrostatic charge in quartz enables the determination of the accumulated net charge on its surface. This procedure involves integrating the electronic density profile, which is displayed in Figure 10. Different scenarios, characterized by various salts and concentrations, have been graphically depicted. In general terms, it was observed that lithium chloride induced the most pronounced screening of surface charges on quartz, as we see in Figure 8. As for trends concerning the other cations, these become less discernible due to the low absorption of polyacrylate in these cases (Figure 3), resulting in the absence of well-defined patterns for the remaining salts. Nevertheless, in all cases, the presence of cations triggers a screening effect on the surface charges.

## 5. Conclusions

In this study, conclusions were obtained from analyzing the effect of dissolved cations in quartz suspensions using both rheology experiments and computer simulations. The key findings of this research are as follows:

- It was confirmed that the yield stress is directly affected by the presence of cations following a specific sequence: Cs > K > Na > Li. This suggests that the adsorbed cations of the “breaker” type have a higher affinity for the quartz surface without the presence of polyacrylate. This observation is related to the weak charge density on the quartz surface, which predisposes it to interact more favorably with low charge density breaking cations.
- The addition of PAA was shown to reduce the yield stress in all cases studied, which indicates its leading role as a dispersant for quartz particles. However, it has been observed that this reduction is more marked in the presence of Li and Na compared to K and Cs. This implies that PA exerts a stronger effect in the presence of Li and Na, suggesting that “maker” ions with PA can overcome the breaker-adsorbed ions in quartz.
- Molecular dynamics simulations supported the experimental results, revealing that the adsorption of the PA polymer on quartz follows the sequence Li > Na > K > Cs. Furthermore, it was observed that higher salt concentrations increase the adsorption of PA on the quartz surface. These results align with the experimental findings, indicating that the presence of cations facilitates PA adsorption.
- Molecular interactions revealed that the formation of cationic bridges was more pronounced on Li and Na, and these bridges contribute to the formation of hydrogen bonds and hydrophobic bridges, increasing adsorption stability on Li and Na. In contrast, the interactions are weak in the cases of K and Cs.
- These results highlight the utility of computational simulations to support experimental observations and provide a deeper understanding of the optimal conditions for admixture rheology modifications in mining processes.

## Data Availability

The data presented in this study are available on request from authors G.R.Q. and M.J.

## Supplementary Materials

The following supporting information can be downloaded at: https://www.mdpi.com/article/10.3390/polym15193861/s1, Figure S1: Fourier-transform infrared spectroscopy (FTIR) of sodium polyacrylate.
