# Supplementary material for: Understanding the Behavior of Sodium Polyacrylate in Suspensions of Silica and Monovalent Salts
Source: Polymers (Basel). 2023 Sep 22;15(19):3861. doi: 10.3390/polym15193861 (PMC10575086; doi:10.3390/polym15193861)
Supplement: Supplementary file 1 [file polymers-15-03861-s001.zip › polymers-2623601-supplementary.pdf]

Supplementary information: Understanding the behavior of sodium polyacrylate in suspensions of silica and monovalent salts

Gonzalo R. Quezada, Francisco Retamal, Matías Jeldres, Ricardo I. Jeldres

Spectrum of sodium polyacrylate

Figure S1 shows the spectrum of NaPA, highlighting the symmetric (1408 cm^{-1}) and antisymmetric (1563 cm^{-1}) stretching of the carboxylate functionality. The spectrum also shows displacement (1327 cm^{-1}) and deformation (1454 cm^{-1}) characteristic of CH_2 and asymmetric stretching (2945 cm^{-1}) characteristic of C-H. The broad band (3203×3386 cm^{-1}) corresponds to stretching vibrations of the hydroxyl group.

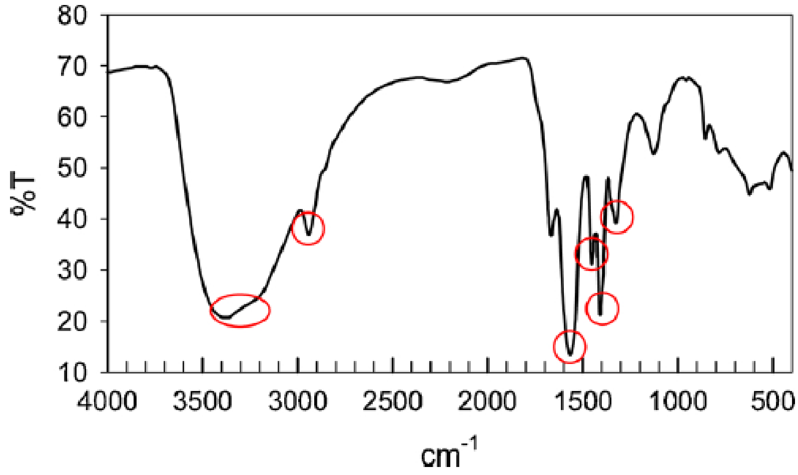

Figure S1. Fourier-transform infrared spectroscopy (FTIR) of sodium polyacrylate.
